# Moxibustion in the management of irritable bowel syndrome: systematic review and meta-analysis

**DOI:** 10.1186/1472-6882-13-247

**Published:** 2013-10-02

**Authors:** Jae-Woo Park, Byung-Hee Lee, Hyangsook Lee

**Affiliations:** 1 Gastroenterology Division, Department of Internal Medicine, College of Korean Medicine, Kyung Hee University, Seoul, Korea; 2 Department of Medical Science of Meridian, Graduate School, Kyung Hee University, Seoul, Korea; 3 Acupuncture & Meridian Science Research Centre, College of Korean Medicine, Kyung Hee University, Seoul, Korea

## Abstract

**Background:**

Irritable bowel syndrome (IBS) is a common functional gastrointestinal disorder. Many patients suffer from IBS that can be difficult to treat, thus complementary therapies which may be effective and have a lower likelihood of adverse effects are being sought.

This systematic review and meta-analysis aimed at critically evaluating the current evidence on moxibustion for improving global symptoms of IBS.

**Methods:**

We searched Medline, EMBASE, the Cochrane Central Register of Controlled Trials, AMED, CINAHL, and CNKI databases for randomised controlled trials (RCTs) of moxibustion comparing with sham moxibustion, pharmacological medications, and other active treatments in patients with IBS. Trials should report global symptom improvement as an outcome measure. Risk of bias for each RCT was assessed according to criteria by the Cochrane Collaboration, and the dichotomous data were pooled according to the control intervention to obtain a risk ratio (RR) of global symptom improvement after moxibustion, with 95% confidence intervals (CI).

**Results:**

A total of 20 RCTs were eligible for inclusion (n = 1625). The risk of bias was generally high. Compared with pharmacological medications, moxibustion significantly alleviated overall IBS symptoms but there was a moderate inconsistency among studies (7 RCTs, RR 1.33, 95% CI [1.15, 1.55], I^2^ = 46%). Moxibustion combined with acupuncture was more effective than pharmacological therapy but a moderate inconsistency among studies was found (4 RCTs, RR 1.24, 95% CI [1.09, 1.41], I^2^ = 36%). When moxibustion was added to pharmacological medications or herbal medicine, no additive benefit of moxibustion was shown compared with pharmacological medications or herbal medicine alone. One small sham-controlled trial found no difference between moxibustion and sham control in symptom severity (mean difference 0.35, 95% CI [−0.77, 1.47]). Moxibustion appears to be associated with few adverse events but the evidence is limited due to poor reporting.

**Conclusions:**

This systematic review and meta-analysis suggests that moxibustion may provide benefit to IBS patients although the risk of bias in the included studies is relatively high. Future studies are necessary to confirm whether this finding is reproducible in carefully-designed and conducted trials and to firmly establish the place of moxibustion in current practice.

## Background

Irritable bowel syndrome (IBS) is a chronic or recurrent functional gastrointestinal (GI) disorder characterised by abdominal pain or discomfort and disturbance of bowel habit for at least three months. Approximately 5 – 20% of adult population suffers from IBS worldwide [1]. IBS is associated with significantly impaired health-related quality of life, and reduce work productivity. Patients with IBS spend health care resources over 1.5 times more than those without IBS [2].

Despite the high prevalence and socioeconomic burden of IBS, the etiology and pathophysiology of IBS remain incompletely understood. Until now, abnormal intestinal motility, visceral hypersensitivity, abnormal neurohormonal responses to stimuli or stress and alteration of normal intestinal microflora are known to be causes of IBS [3]. Therefore, recent conventional treatments such as antispasmodics, fiber supplementation and antidepressants have focused on the alleviation (or relief) of intestinal IBS symptoms, however limited effects of them have made many IBS patients interested in complementary and alternative medicine (CAM) [4]. Particularly, acupuncture-related interventions are one of the most frequently sought CAM modalities and have been widely used in various conditions including functional GI disorders with 12 million treatments per year in the US [5,6].

Of acupuncture-related techniques, moxibustion or moxa, frequently used in conjunction with acupuncture needling, uses heat stimulation generated by burning herbal preparations containing dried *Artemisia vulgaris* or mugwort leaves on targeted acupuncture points to improve general health and treat chronic conditions such as arthritis and digestive disorders [7]. It is generally classified into direct and indirect moxibustion; while heat stimulation is applied directly to the skin surface in direct moxibustion often inducing pain and scarring, various insulating materials such as ginger, salt, or herbal cake, are placed between the burning moxa cone and the skin surface, or moxa cone is burnt on top of an acupuncture needle that has been left in place in indirect moxibustion [7]. Moxibustion has been widely used for various conditions including cancer, ulcerative colitis, stroke rehabilitation, constipation, hypertension, pain conditions and breech presentation [8].

A recent systematic review on acupuncture for IBS has concluded that acupuncture is better than conventional medications in improving global IBS symptoms but further studies are warranted to clarify the reported greater benefits of acupuncture relative to medications are due to patients’ preference to acupuncture or expectations [9]. Although moxibustion is frequently used for IBS in practice, with acupuncture or separately, there has been no systematic study to inform current evidence on effectiveness of moxibustion treatment for IBS. It would be helpful for decision-making for patients with IBS who do not respond well to conventional treatments if we could provide currently available evidence for or against moxibustion in IBS.

Therefore, we conducted a systematic review and meta-analysis to summarise and critically evaluate all of the currently available randomised controlled trials (RCTs) of moxibustion comparing with sham moxibustion, pharmacological medications or other active treatments for symptom improvement in patients with IBS.

## Methods

### Data sources

We searched the Cochrane Central Register of Controlled Trials (inception to August 2012), Ovid Medline (1946 to August 2012), Ovid EMBASE (1980 to February 2011), the Allied and Complementary Medicine Database (AMED, 1990 to August 2012), the Cumulative Index to Nursing and Allied Health Literature (CINAHL, 1996 to August 2012), and China National Knowledge Infrastructure databases (CNKI, 1994 to August 2012). Reference lists of reviews and relevant articles were screened for additional studies.

Search terms used for Cochrane Central Register of Controlled Trials were as follows: (irritable OR functional OR spastic) AND (bowel OR colon) for IBS search and (acupunctur* OR electroacupuncture OR electro-acupuncture OR acupoint OR meridian OR auriculoacupuncture OR moxa OR moxibustion*) for moxibustion search were combined. These search terms were slightly modified for other databases. Trials published in English, Korean and Chinese were sought considering countries in which moxibustion has been widely used.

### Study selection

RCTs comparing the effect of moxibustion with sham moxibustion, no treatment, or other treatments such as pharmacological medications in patients with IBS (aged > 16 years) were eligible for inclusion. Moxibustion combined with related techniques such as acupuncture was allowed. Trials that allowed other concomitant treatments were eligible, as long as they were given to both the moxibustion and control groups. Studies comparing moxibustion with Chinese herbal medicine or other types of moxibustion of which the effect has not been established were excluded. The first period of crossover RCTs was also considered. The diagnosis of IBS could be based on either a clinician’s opinion, or specific diagnostic criteria (Manning, Kruis score, or Rome I, II, or III), supplemented by the negative GI investigations to exclude organic diseases. The primary outcome was improvement of global IBS symptoms or overall IBS symptom scores after completion of treatment reported as a dichotomous or continuous variable.

### Data extraction and risk of bias assessment

Two reviewers (Jae-Woo Park & Byung-Hee Lee) independently reviewed all searched articles to evaluate suitability for inclusion. If there was disagreement, it was resolved by discussion with the corresponding reviewer (Hyangsook Lee) and further information was sought from the relevant sources [9,10]. After selection of studies, the aforementioned two reviewers extracted data from the selected articles independently: author, year of publication, country of origin, study design, participants (sample size, proportion of female participants), diagnostic criteria used to define IBS, type of IBS based on the predominant stool form, outcome measures used to define global symptom improvement or cure following treatment, moxibustion intervention, control intervention, main results and adverse events. Data were extracted as intention-to-treat analyses, i.e. all withdrawals and dropouts were assumed to be treatment failures, if trial reporting provided relevant information. Although including participants who have not completed treatment in the analysis as treatment failures is likely to underestimate the effect of moxibustion, it can be the more conservative and rigorous analytical approach.

Risk of bias for the included studies was evaluated by the two reviewers (Jae-Woo Park & Byung-Hee Lee) according to the Cochrane Collaboration’s risk of bias assessment tool [11]. The criteria refer to characteristics of the study that might be related to selection bias (random sequence generation and allocation concealment), performance bias (blinding of participants and personnel), detection bias (blinding of outcome assessment), attrition bias (incomplete outcome data), and reporting bias (selective outcome reporting) [11]. Each criterion was scored as yes (Y), no (N), or unclear (U), where yes indicates low risk of bias, no indicates high risk of bias, and unclear indicates uncertain risk of bias. Disagreements were resolved by discussion.

### Data synthesis and statistical analysis

Data were pooled using a random effects model as we considered the treatment effects for the individual studies would vary due to expected variability in moxibustion interventions, control procedures, outcome measures, and methodological quality. Studies were classified and combined in the analysis according to the outcome measure, intervention type, and/or control intervention. The impact of moxibustion on dichotomous outcomes such as global IBS symptom improvement at the end of treatment was expressed as risk ratio (RR) with 95% confidence intervals (CI), while the effect of moxibustion on continuous outcomes such as IBS symptom scores was examined using mean difference (MD) with 95% CI. Review Manager Software (version 5.1 for Windows; The Nordic Cochrane Centre, Copenhagen, Denmark) was used to generate forest plots of pooled RRs and MDs with 95% CI. A chi-square test with a significance level of *p* < 0.1 was used to assess heterogeneity. To quantify inconsistencies among the included studies, the I^2^ test was used. The I^2^ statistic indicates the proportion of variability across studies not explained by chance alone and the I^2^ value of 50% or more was considered to be an indicator of substantial level of heterogeneity [12,13].

As a sensitivity analysis, we evaluated whether the findings were affected if we excluded studies with a high/unclear risk of bias for randomisation and/or allocation concealment [14,15], or if we assumed missing data as failed cases in the moxibustion group and put them as such in our re-analysis. During data extraction, we found that not all trials in this review adopted evidence-based pharmacological medications [16]. Therefore, as a post-hoc analysis, we examined whether the estimate had any difference between all trials and trials adopting controls of evidence-based pharmacological medications.

We also explored by subgroup analyses whether the treatment effect was affected according to the type of IBS based on the predominant stool type, or gender. These are observational by nature and are not based on randomised comparisons [12], thus the results should be interpreted with caution.

## Results

### Selection and characteristics of eligible studies

**Figure 1:**
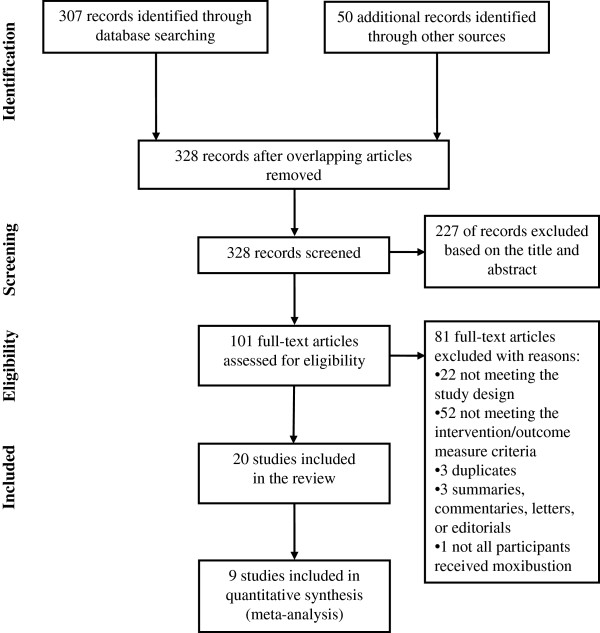
Flow diagram of literature search.

The search generated 328 citations, of which 101 full-text articles were read for further eligibility assessment. Of these 101 articles, 81 were excluded; leaving 20 eligible RCTs involving 1625 participants in the systematic review [17-36]. Figure 1 shows a flowchart of literature searching as in the Preferred Reporting Items for Systematic Reviews and Meta-Analyses (PRISMA) [37]. A PRISMA checklist [37] is also available as an Additional file 1. Except for one American study [18], all were conducted in China as comparative effectiveness trials. Participants had diarrhoea-predominant type (12 trials), constipation-predominant type (2 trials) and 6 studies did not specify IBS type. Rome II or III criteria, traditional Chinese Medicine diagnostic criteria, and/or negative GI investigations were used for diagnosis. For moxibustion intervention, 8 trials tested moxibustion alone [20,22,25-28,32,36]; moxibustion combined with acupuncture was used in 7 studies [17-19,21,31,34,35]; moxibustion and pharmacological medication was used in two studies [23,29]; two studies used moxibustion with herbal medicine [30,33] and one used moxibustion with psychotherapy [24]. Moxibustion treatment period ranged from 10 to 75 days (median 30 days) and was given once daily in 80% of the included studies. Various controls were used; pharmacological medications in 14 trials [19,20,22,23,25-29,31,32,34-36], herbal medicine in two trials [30,33], and sham moxibustion [18], probiotics [17], acupuncture [21], and psychotherapy [24] in one trial each. Improvement in global IBS symptoms was reported using a 3 or 4-point Likert type scale in 18 trials [17,19-27,29-36]; one study used pre-defined IBS symptom score in total [28], and another study used a 7-point Likert type clinical global impression scale [18] (Tables 1 and 2).

**Table 1:** Characteristics of randomised controlled trials of moxibustion vs. pharmacological medications for IBS

**Study (year)** **Country**	**Sample size (% female)**	**Diagnostic criteria used for IBS**	**Type of IBS (based on the predominant stool form)**	**Criteria used to define symptom improvement**	Moxa intervention (duration)*	**Control intervention (duration)**	**Risk of bias assessment**^**a**^
Luo (2012) China [26]	40 (53%)	Rome III, negative GI investigations and TCM criteria (liver-qi stagnation type)	C 100%	Any improvement in global IBS symptoms	Moxa (4 weeks): Herbal cake-partitioned and individualised, o.d.	Medication (4 weeks): Mosapride 5 mg/time, t.i.d.	U-U-N-N-Y-Y
Chu (2011) China [20]	60 (22%)	Rome II and TCM criteria	D 100%	≥ 30% improvement in global IBS symptoms	Moxa (15 days): Indirect and partially individualised, o.d.	Medication (15 days): Loperamide 2 mg/time, b.d.	Y-U-N-N-Y-Y
Luo (2011) China [25]	60 (42%)	Rome III, IBS-C according to Bristol Stool Form Scale, and negative GI investigations	C 100%	≥ 30% improvement in global IBS symptoms	Moxa (2 weeks): Indirect and fixed, o.d.	Medication (2 weeks): Mosapride 5 mg/time, t.i.d.	U-U-N-N-Y-Y
Luo (2008) China [27]	95 (49%)	Rome III, negative GI investigations and Standards for clinical diagnosis for IBS from 1986 National conference for chronic diarrhea	D 100%	Any improvement in global IBS symptoms	Moxa (30 days): Indirect and fixed, b.d., 10 days/course, 3 courses in total	Medication (30 days): Pinaverium, 50 mg/time, t.i.d.	U-U-N-N-Y-Y
Huang (2007) China [22]	65 (unspecified)	Rome III, negative GI investigations and TCM criteria	Unspecified	Any improvement in global IBS symptoms	Moxa (4 weeks): Indirect and partially individualized, o.d.	Medication (4 weeks): Trimebutine 0.2 g/time, t.i.d.	U-U-N-N-Y-Y
Zhang (2007) China [36]	60 (62%)	Rome II	D 100%	≥ 30% improvement in global IBS symptoms	Moxa (2 weeks): Ginger-partitioned and fixed, o.d. for 2 weeks	Medication and standard care (2 weeks):	U-U-N-N-Y-Y
						- Standard care such as diet, psychiatric, and anti-diarrheal therapy	
						- Entero-soluble glutamine 0.4 g, t.i.d. or smecta 3 g, t.i.d. or probiotics 630 mg, t.i.d.	
Ni (2001) China [28]	56 (63%)	Negative GI investigations and Standards for clinical diagnosis for IBS from 1986 National conference for chronic diarrhea	D 100%	Change of total IBS symptom score (pre-defined)	Moxa (15 days): Indirect and partially individualized, o.d. for 15 days	Medication (15 days): Nifedipinum, 10 mg/time, t.i.d.	U-U-N-N-N-N
Wu (1996) China [32]	81 (44%)	Standards for clinical diagnosis for IBS from 1986 National conference for chronic diarrhea and TCM criteria	Unspecified	Any improvement in global IBS symptoms	Moxa (72 days): Herbal cake-partitioned and individualised, o.d., 12 sessions/course, 5 courses in total, with 3 days of no TX interval	Medication (3 months):	U-U-N-N-Y-Y
						- Piperazine 0.2 g/time, t.i.d.	
						- Smecta, 3 g/time, t.i.d.	

^a^Risk of bias was evaluated for 6 criteria in order [11], i.e. sequence generation, allocation concealment, blinding of participants, blinding of outcome assessors, incomplete outcome data, and selective outcome reporting. Each criterion was scored as yes (Y), no (N), or unclear (U), where Y indicates a low risk of bias, N indicates a high risk of bias and U indicates an unclear risk of bias.

*Moxibustion method was classified into three categories on the basis of the levels of individualisation: “fixed” means all patients receive the same treatment at all sessions, “partially individualised” means using a fixed set of points to be combined with a set of points to be used flexibly, and “individualised” means each patient receives a unique and evolving diagnosis and treatment [38].

b.d., twice a day; C, constipation-predominant subtype of IBS; D, diarrhoea-predominant subtype of IBS; GI, gastrointestinal; IBS, irritable bowel syndrome; moxa, moxibustion; o.d., once a day; TCM, traditional Chinese Medicine; t.i.d., three times a day; TX, treatment.

**Table 2:** Characteristics of randomised controlled trials of moxibustion vs. sham or other treatments for irritable bowel syndrome

**Study (year) Country**	**Sample size (% female)**	**Diagnostic criteria used for IBS**	**Type of IBS (based on the predominant stool form)**	**Criteria used to define symptom improvement**	Moxa intervention (duration)*	**Control intervention (duration)**	**Risk of bias assessment**^**a**^
**Moxa/AT vs. sham moxa/AT**							
Anastasi (2009) USA [18]	29 (66%)	Rome III and negative GI investigations	Unspecified	Changes in CGIS	Moxa and AT (4 weeks): Indirect and individualised, twice weekly	Sham moxa and sham AT (4 weeks): Superficial needling at non-acupoints/moxa above and away from acupoints	U-U-Y-Y-U-Y
**Moxa/AT vs. pharmacological medications**							
Chen (2011) China [19]	59 (53%)	Rome III, negative GI investigations and TCM criteria	D 100%	≥ 30% improvement in global IBS symptoms	Moxa and AT (3 weeks): Indirect and partially individualised, o.d., 5 sessions/course, 3 courses in total	Medication (3 weeks):	Y-Y-N-N-Y-Y
						- Smecta 1 bag/time b.d.	
						- Loperamide 4 mg/time t.i.d. and pinaverium bromide 50 mg/time t.i.d. if diarrhea did not stop	
Zeng (2010) China [35]	65 (58%)	Rome III	D 100%	≥ 50% improvement in global IBS symptoms	Moxa and AT (1 month): Indirect and partially individualized, o.d., 10 sessions/course in dog days	Medication (1 month): Trimebutine maleate 100 mg/time, t.i.d.	Y-Y-N-N-Y-Y
Xue (2009) China [34]	200 (51%)	Rome II and TCM criteria	Unspecified	Any improvement in global IBS symptoms	Moxa and AT (23–49 days): Fixed, o.d. for 10 sessions (1 course), 2–4 courses in total, with 3 days of no TX interval	Medication (23–49 days): Sulfasalazine 10 mg/kg, o.d. for 10 days (1 course), 2–4 courses in total	U-U-N-N-Y-Y
Wang (2008) China [31]	110 (unspecified)	Rome II	D 100%	≥ 30% improvement in global IBS symptoms	Warming needle (23 days): Fixed, o.d. for 10 sessions (1 course), 2 courses in total, with 3 days of no TX interval	Medication (23 days): Smecta, 1 bag/time t.i.d.	U-U-N-N-Y-Y
**Moxa plus other treatments vs. other treatments**							
Hu (2012) China [21]	64 (22%)	Rome III, negative GI investigations and TCM criteria	D 100%	≥ 30% improvement in global IBS symptoms	Moxa and AT (8 weeks):	AT (8 weeks): Partially individualized, o.d., 5 sessions/week, 20 sessions/course, 2 courses in total	Y-U-N-N-Y-Y
					- Moxa: indirect and partially individualized, o.d., 5		
					sessions/week, 20 sessions/course, 2 courses in total		
					- AT: partially individualized, o.d., 5 sessions/week, 20 sessions/course, 2 courses in total		
Shang (2012) China [29]	48 (58%)	Rome II and negative GI investigations	D 100%	Any improvement in global IBS symptoms	Moxa/AT and medication (30 days): Indirect and fixed, o.d., 10 sessions/course, 3 courses in total	Medication and dietary advice (30 days):	U-U-N-N-Y-Y
						- GI antispasmodic drugs, antidiarrheal drugs, anti-anxiety drugs, and intestinal flora regulating drugs	
Jiang (2010) China [23]	60 (57%)	Rome II and negative GI investigations	D 100%	Any improvement in global IBS symptoms	Moxa and medication (4 weeks):	Medication (4 weeks): Trimebutine 0.2 g/time, t.i.d.	U-U-N-N-U-N
					- Moxa: ginger-partitioned and fixed, o.d. for 7 sessions (1 course), 4 courses in total, with 1 day of no TX interval		
					- Medication: Trimebutine 0.2 g/time, t.i.d.		
Wang (2009) China [30]	60 (55%)	Rome II and TCM criteria (liver-qi stagnation with spleen deficiency type)	D 100%	Any improvement in global IBS symptoms	Moxa and herbal medicine (1 month):	Herbal medicine (1 month): Partially individualised	U-U-N-N-Y-Y
					- Moxa: indirect and fixed, o.d.		
					- Herbal medicine: partially individualised		
Xiong (2008) China [33]	120 (60%)	Rome II, negative GI investigations and TCM criteria (liver-qi stagnation with spleen deficiency type)	D 100%	≥ 30% improvement in global IBS symptoms	AT/warming needle and herbal medicine (4 weeks):	Herbal medicine (4 weeks): Fixed, b.d.	U-U-N-N-Y-Y
					- AT/warming needle: fixed, no information on sessions		
					- Herbal medicine: fixed, b.d.		
Huang (2007) China [22]	61 (unspecified)	Rome III, negative GI investigations and TCM criteria	Unspecified	Any improvement in global IBS symptoms	Moxa and colon hydrotherapy (4 weeks):	Colon hydrotherapy (4 weeks): Twice weekly for constipation, once weekly for diarrhea	U-U-N-N-Y-Y
					- Indirect and partially individualized, o.d.		
					- Colon hydrotherapy: twice weekly for constipation, once weekly for diarrhea		
Liu (1997) [24] China	150 (41%)	Only stated that “all participants had IBS and no organic GI disease”	Unspecified	Any improvement in global IBS symptoms	AT/moxa and psychotherapy (10–75 days): Individualised AT followed by indirect and fixed moxa, once every other day for 10 sessions (1 course), 1–6 courses, with 3–5 days of no TX interval	Psychotherapy (7–42 days): 1–2 sessions/week (1 course), 1–6 courses, each session performed ahead of AT	Y-U-N-N-N-Y
**Moxa/AT vs. probiotics**							
An (2010) China [17]	81 (62%)	Rome II	Unspecified	≥ 30% improvement in global IBS symptoms	Moxa and AT (4 weeks): Indirect and fixed, o.d. for 12 sessions (1 course), 2 courses in total, with 3 days of no TX interval	Bifid triple viable capsules (4 weeks): *Bifidobacterium longum, Lactobacillus acidophillus, and Enterococcus faecalis* 420 mg (2 capsules)/time, t.i.d.	Y-U-N-N-Y-Y

^a^Risk of bias was evaluated for 6 criteria in order [11], i.e. sequence generation, allocation concealment, blinding of participants, blinding of outcome assessors, incomplete outcome data, and selective outcome reporting. Each criterion was scored as yes (Y), no (N), or unclear (U), where Y indicates a low risk of bias, N indicates a high risk of bias and U indicates an unclear risk of bias.

*Moxibustion method was classified into three categories on the basis of the levels of individualisation: “fixed” means all patients receive the same treatment at all sessions, “partially individualised” means using a fixed set of points to be combined with a set of points to be used flexibly, and “individualised” means each patient receives a unique and evolving diagnosis and treatment [38].

AT, acupuncture; b.d., twice a day; CGIS, 7-point Likert-type Clinical Global Impression Scale; D, diarrhea-predominant subtype of IBS; GI, gastrointestinal; GP, general practitioner; IBS, irritable bowel syndrome; moxa, moxibustion; o.d., once a day; SSS, severity scoring system; TCM, traditional Chinese medicine; t.i.d., three times a day; TX, treatment.

### Risk of bias in the included studies

Six studies reported adequate methods for sequence generation [17,19-21,24,35], of which two studies also did so for allocation concealment [19,35]. As all Chinese comparative effectiveness trials compared moxibustion with other active treatment, or evaluated moxibustion as an adjunct to other treatment given to all study participants, participant blinding was impossible thus outcome assessment, i.e. assessment of improvement in global IBS symptoms by participants was not blinded in the 19 included studies. This is likely to have introduced the main risk of bias in these studies. One study was evaluated as having a high risk of bias for incomplete outcome data and selective outcome reporting as it provided insufficient reporting of number of participants analysed for improvement in global IBS symptoms outcome, thus could not be entered into a pooling [28]. Some studies were also rated as having a high/unclear risk of bias for incomplete outcome data or selective outcome reporting as they did not report the exact number of participants in their analyses [23,24] or they used other method that was not pre-defined in the outcome assessment methods [23] (Table 1; Additional files 2 and 3).

### Effect of moxibustion on IBS symptom improvement/scores

#### Moxibustion vs. pharmacological medications (8 trials)

**Figure 2:**
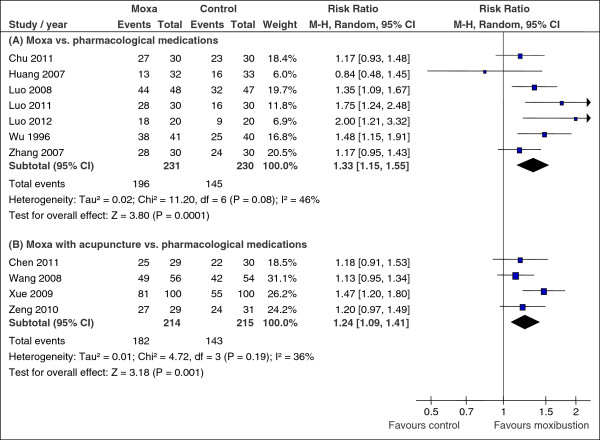
**Moxibustion vs. pharmacological medications. A**, Moxibustion vs. pharmacological medications; **B**, moxibustion plus acupuncture vs. pharmacological medications. All studies assessed improvement in global IBS symptoms, i.e. moxibustion was tested against control procedures by comparing the number of participants who had shown any improvement in IBS symptoms. Vertical line indicates no effect point. CI, confidence interval; IBS, irritable bowel syndrome; moxa, moxibustion.

Moxibustion alone was compared with pharmacological medications in 8 studies [20,22,25-28,32,36]. Of these studies, 7 studies [20,22,25-27,32,36] with 461 participants reported the number of participants whose global IBS symptoms improved at the end of treatment (median 4 weeks) as a dichotomous outcome, i.e. response rate (Table 1). Moxibustion had a statistically significant effect in improving IBS symptoms compared with pharmacological medications with moderate inconsistency across trials (RR of any symptom improvement = 1.33, 95% CI [1.15, 1.55], χ^2^ = 11.20, degrees of freedom (df) = 6, *p* = 0.08, I^2^ = 46%, Figure 2. (A)). One trial with a low risk of bias for randomisation [20], however, detected no statistically significant difference (RR 1.17, 95% CI [0.93, 1.48]). One study did not report the number of participants whose IBS symptoms improved [28]; instead, improvement rate based on total symptom score changes was reported for each group (n = 56; 79.3% in the moxibustion group vs. 64.0% in the pharmacological medication group). When we restricted pooling to two trials [22,27] comparing moxibustion with evidence-based antispasmodics [16] in a post-hoc sensitivity analysis, the results did not favour moxibustion (RR 1.13, 95% CI [0.69, 1.84], χ^2^ = 2.97, df = 1, *p* = 0.08, I^2^ = 66%). Subgroup analyses of treatment effect were conducted according to the IBS type; three studies [20,27,36] only involving patients with diarrhoea-predominant IBS reported statistically significant benefit from moxibustion compared with pharmacological medications (RR 1.23, 95% CI [1.08, 1.39], χ^2^ = 1.14, df = 2, *p* = 0.56, I^2^ = 0%). Likewise, moxibustion improved global symptoms of IBS significantly more than pharmacological medications in constipation-predominant IBS patients (two trials [25,26], RR 1.83, 95% CI [1.37, 2.43], χ^2^ = 0.18, df = 1, *p* = 0.67, I^2^ = 0%). No study reported the treatment effect according to gender, and subgroup analysis was not possible for this characteristic.

#### Moxibustion plus acupuncture vs. pharmacological medications (4 trials)

There were 434 participants included in the 4 studies of moxibustion plus acupuncture vs. pharmacological medications [19,31,34,35]. Moxibustion in addition to acupuncture statistically significantly improved global IBS symptoms at the end of median 23-day treatment (RR 1.24, 95% CI [1.09, 1.41], χ^2^ = 4.72, df = 3, *p* = 0.19, I^2^ = 36%, Figure 2. (B)). When pooling was limited to trials with a low risk of bias for randomisation/allocation concealment [19,35], the benefit of moxibustion plus acupuncture in improving IBS symptoms still remained significant (RR 1.19, 95% CI [1.01, 1.41], χ^2^ = 0.02, df = 1, *p* = 0.89, I^2^ = 0%). A post-hoc sensitivity analysis including two RCTs [19,35] testing moxibustion plus acupuncture against evidence-based antispasmodics [16] still favoured moxibustion plus acupuncture (RR 1.19, 95% CI [1.01, 1.41], χ^2^ = 0.02, df = 1, *p* = 0.89, I^2^ = 0%). When we re-analysed the data by including the 4 missing cases in the moxibustion group as failed cases and one in the control group as success in Zeng et al. study [35], the estimate remained significant but there was a substantial heterogeneity among studies (RR 1.20, 95% CI [1.03, 1.40], χ^2^ = 6.23, df = 3, *p* = 0.10, I^2^ = 52%). There also was a treatment benefit from moxibustion plus acupuncture for diarrhoea-predominant IBS patients compared with pharmacological medications (three studies [19,31,35], RR 1.16, 95% CI [1.03, 1.31], χ^2^ = 0.24, df = 2, *p* = 0.89, I^2^ = 0%). No study reported the treatment effect according to gender.

#### Moxibustion as an adjunct to other treatments (7 trials)

Seven trials involving 556 participants tested moxibustion plus other treatments against other treatment alone [17,21-24,29,30,33]. Other treatments included acupuncture, pharmacological medications, herbal medicine, colon hydrotherapy and psychotherapy. They all reported on improvement in global IBS symptoms at the end of median 4-week treatment.

As the studies were clinically diverse in terms of intervention and control, we did not combine them. Instead, we classified studies based on control group. Two studies [23,29] investigated the effect of moxibustion as an add-on treatment to pharmacological medication and no significant benefit was detected (RR 1.19, 95% CI [0.81, 1.74]). However, there was a considerable heterogeneity (χ^2^ = 3.99, df = 1, *p* = 0.05, I^2^ = 75%). Only one study [23] tested moxibustion as an adjunct to evidence-based antispasmodic drug, trimebutine [16], and no additive benefit was demonstrated (RR 1.04, 95% CI [0.89, 1.21]). When moxibustion in addition to herbal medicine was compared with herbal medicine alone, there was no significant effect of moxibustion (RR 1.14, 95% CI [1.00, 1.29], χ^2^ = 0.12, df = 1, *p* = 0.73, I^2^ = 0%). The remaining three studies tested moxibustion as an adjunct to acupuncture [21], colon hydrotherapy [22], and psychotherapy [24], respectively. Of them, there was a significant benefit of moxibustion when added to colon hydrotherapy (RR 1.57, 95% CI [1.09, 2.27]) or psychotherapy (RR 1.20, 95% CI [1.03, 1.39]).

#### Moxibustion plus acupuncture vs. probiotics (1 trial)

An et al. compared 4-week moxibustion and acupuncture treatment with bifid triple viable capsules three times a day for 4 weeks [17]. They did not find any benefit of moxibustion and acupuncture as an add-on to probiotics (RR 1.14, 95% CI [0.92, 1.42]).

#### Moxibustion plus acupuncture vs. sham moxibustion plus sham acupuncture (1 trial)

**Figure 3:**
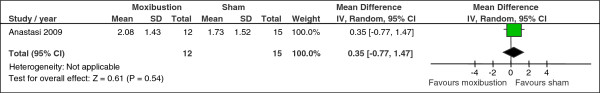
**Moxibustion vs. sham moxibustion.** Moxibustion in addition to acupuncture was tested against sham moxibustion and sham acupuncture using the 7-point Likert type Clinical Global Impression Scale. Vertical line indicates no effect point. CI, confidence interval.

Anastasi et al. (2009) tested twice weekly moxibustion treatment in addition to acupuncture against sham moxibustion and sham acupuncture [18]. This study did not find any significant between-group difference measured with the 7-point Likert-type Clinical Global Impression Scale (MD 0.35, 95% CI [−0.77, 1.47], Figure 3.).

### Adverse events associated with moxibustion

A total of 4 RCTs reported on adverse events associated with moxibustion [18,22,23,31]. Of these 4 trials, three trials reported that there were no adverse events [18,22,31]. Jiang et al. reported that two out of 30 participants in the moxibustion combined with pharmacological medication group felt thirsty probably due to medication but completed treatment [23].

## Discussion

### Summary of main findings

Currently, patients with IBS have few effective treatments available or some modestly effective treatments are not entirely free from risks [2], thus safe and effective nonpharmacological treatments for IBS have been sought. This systematic review and meta-analysis has shown that moxibustion has a therapeutic benefit in improving global symptoms of IBS. In 7 Chinese comparative effectiveness trials, moxibustion was better than pharmacological medications in improving global symptoms of IBS, but this needs to be carefully interpreted given moderate inconsistency detected. Four trials found that participants receiving moxibustion plus acupuncture reported greater improvement in IBS symptoms compared with those given pharmacological medications. When moxibustion was added as an adjunct to other treatments, it significantly reduced symptoms of IBS relative to other treatment alone. Moxibustion was not effective compared with sham moxibustion but it was based on one small trial. Moxibustion appears to be associated with few adverse events but the data are limited as they are based on reporting from 4 out of 20 studies.

### Applicability of the evidence

Because of the nature of individualised or complex intervention, it is difficult to determine whether investigated treatment is the optimum moxibustion strategy for IBS. Furthermore, as moxibustion is usually given with acupuncture needling or other active treatments in practice, the effect estimate of moxibustion may not be solely attributable to moxibustion alone despite our efforts to extract its own effect by carefully including the trials where other concomitant treatments were given to both moxibustion and control group. As was often the case with acupuncture trials, the moxibustion treatments investigated in the included trials were highly variable. There are various components that would constitute a therapeutic effect of moxibustion, e.g. methods of moxibustion procedures, number of moxibustion sessions, and frequency and duration of moxibustion treatment. All included trials used indirect moxibustion, i.e. various insulating materials such as ginger, salt, or herbal cake, are placed between the burning moxa cone and the skin surface; in 80% of the included studies, participants were given *daily* moxibustion treatment; treatment duration varied but participants received median 30 days of moxibustion treatment. Nineteen out of 20 included studies were conducted in China, and the investigated moxibustion interventions in these trials are then likely to well reflect common moxibustion practice in China [9]. Nevertheless, the moxibustion intervention tested here may not be a generalisable treatment schedule outside China; the only sham-controlled study [18] adopted twice weekly treatment and this was conducted in America. Treatment frequency of twice per week may be seen as insufficient from a Chinese viewpoint.

Although this systematic review indicates the possible benefit from moxibustion for IBS, careful interpretation of the results is needed. Evidence from 7 Chinese comparative effectiveness trials has shown that moxibustion is better than pharmacological medications. As these studies were neither designed to investigate noninferiority nor conducted based on a formal sample size calculation, we cannot be sure whether the findings of no significant difference indicate evidence of equivalence. In addition, these studies cannot avoid performance or detection bias which threatens validity of the results. Participants may have preferred moxibustion to pharmacological medications or cultural backgrounds where patients are more familiar with acupuncture and moxibustion compared to other Western countries may have played a role in positive results. All these should be considered before we make any judgment or recommendation on moxibustion for IBS patients.

### Risk of bias in the included studies

We used the risk of bias assessment tool from the Cochrane Handbook to evaluate the quality of the included studies. The definition of a study with a high or low risk of bias may vary depending on the intervention and condition under scrutiny. In IBS trials, it is also important to comply with the guidelines for the design of intervention studies for functional GI disorders by the Rome committee [39]. In this context, most of the included studies in this review are not entirely free from biases in various aspects. First of all, randomisation and allocation concealment are key factors that may influence the outcome [15,40]. For adequate random sequence generation, unclear risk of bias given to 30% of the included studies [17,19-21,24,35], mostly due to a lack of or poor reporting of randomisation method, raises the possibility that the randomisation process may have been inappropriate [9]. Group assignment was adequately concealed in only 10% of the included studies [19,35] and the rest of the studies were given unclear risk of bias due to a lack of related reporting. Allocation concealment minimises selection and allocation biases by safeguarding the allocation sequence before and until the participants have been allocated and this can be achieved in any trials. On the other hand, blinding safeguards the allocation sequence after randomisation and cannot always be achievable [41]. Then, failure to reporting adequate allocation concealment in most of the included studies of our review poses a question whether the results may have been too optimistic than they should be.

As IBS is a condition which is well recognised to show a high placebo response to treatment [42], ensuring participant and outcome assessment blinding is a critical issue. However, participant blinding in moxibustion studies may neither be feasible nor scientifically meaningful when the specific components to control for in the sham moxibustion group are not well investigated [43]. Furthermore, the patient’s assessment of symptoms at the end of the treatment is a desirable outcome in intervention trials of functional GI disorders [39], which makes outcome assessment blinding more difficult. Failure to blinding may have led to a greater benefit of moxibustion procedures than that of simple medications. There was only one sham-controlled trial [18] out of 20 included studies and the effect of moxibustion against pharmacological medications or combined effect of moxibustion with other treatment compared with other treatment alone may have been overestimated. All these limitations should be taken into account when interpreting the present findings.

### Limitations of this review

Language restrictions in the systematic review can have a different impact on the overall estimate of treatment effects depending on whether the intervention under scrutiny belongs to conventional medicine or CAM [44]. Therefore, efforts should be made for comprehensive literature search to include an unbiased sample of all the relevant studies irrespective languages. We tried to identify all the relevant trials in a range of databases including conventional and specialised databases and also hand-searched reference lists of relevant reviews and articles for additional studies. Although we are confident that our search strategies located all the relevant studies, there is always some degree of uncertainty. In addition, 95% of the included studies were conducted in China where negative studies are seldom published [45,46] and our review may have been affected by the potential publication bias. A recent Health Technology Assessment report has found that including non-English articles or studies published in journals which are not indexed in Medline in a meta-analysis increases the degree of asymmetry in the funnel plot [47]. It may be pertinent to our review, but the number of pooled studies was too small to formally test for funnel plot asymmetry. Future trials would allow us to detect small-study effects in this area.

Systematic reviews and meta-analyses are often limited by the quality of the included studies. The quality of the present evidence is limited considering that most of the included studies were given unclear risk of bias for key methodological elements of adequate random sequence generation and allocation concealment. In addition to this, high risk of bias associated with patient/outcome assessment blinding also should be considered as the effect of moxibustion has been likely to be estimated higher than it actually is. It is also worth noting that the treatment effect may have been affected when the results in the included studies were largely dependent on measurement by Likert-type scale where social desirability or central tendency bias might have come into play.

Another limitation of this review is the lack of data reported by the trials, which restricted our subgroup analyses. Subgroup analyses according to the participant’s predominant stool type found moxibustion is beneficial to both diarrhoea-predominant and constipation-predominant types, but these analyses are observational, thus the evidence is limited. Especially, we could not examine the effect of moxibustion according to the gender due to the lack of data reporting.

Last but not the least, investigating the sources of heterogeneity as well as measuring inconsistency of the results across studies is a critical part of meta-analysis [48,49]. Both clinical diversity in interventions, patient characteristics or choice of outcome measures, and methodological diversity such as implementation of allocation concealment or blinding can lead to statistical heterogeneity. Although a level of significance was set at 10% rather than 5% in our statistical test for heterogeneity, chi-square test in our review has low power and in the I^2^ test, preferable to the test of heterogeneity, an I^2^ value of 50% as an indicator of substantial heterogeneity can only be a rough guide [12,49]. In our pre-specified subgroup analyses for investigating heterogeneity, we could not make valid comparisons between types of IBS due to a small number of studies. All these limitations increase the difficulty in drawing overall conclusions.

### Implications for practice and research

Patients with IBS have few effective treatment options and tend to feel stigmatised that they are labelled as neurotic by doctors despite their intractable symptoms [50]. Evidence-based pharmacological medications have modest efficacy [51] and even doctors experience frustration with IBS, due as much to medical uncertainty and shortage of effective interventions as to intolerance of the personal characteristics of IBS patients [50]. Effective and safe complementary treatments may thus be attractive to patients failing to respond to conventional medications and doctors treating them.

Although this systematic review and meta-analysis suggests that moxibustion may provide benefit to patients with IBS, there are several issues to be considered before recommending it to patients. As mentioned above, the tested intervention may not be the optimum moxibustion strategy for IBS. In addition, we cannot be sure how treatment strategy should differ for different types of IBS patients. Although our subgroup analyses supported moxibustion for diarrhoea-predominant IBS compared with pharmacological medication and with other therapy, and also for constipation-predominant IBS compared with pharmacological medication, these analyses are all observational by nature and based on a small number of studies. Future trials should also be able to answer questions regarding adequate moxibustion method and dosage, treatment frequency and duration, and treatment period. The majority of the included studies tested mean 30 sessions of daily moxibustion treatment and it should be tested whether such interventions are applicable in different contexts. It should be noted that not all trials in this review adopted evidence-based pharmacological therapy as a comparator and the present evidence, therefore, may be incomplete.

Moxibustion shares characteristics in common with acupuncture. Therefore, it would help interpret the present results to consider the findings from a recent systematic review of acupuncture for IBS [9]; in their review, high-quality, sham-controlled trials found no benefits of acupuncture relative to a credible sham control, while patients reported greater benefits from acupuncture than from pharmacological medications in comparative effectiveness trials. In our review, we also found greater benefit from moxibustion than from pharmacological medication while a sham-controlled trial did not favour real moxibustion over sham control [18]. As we only had one pilot sham-controlled trial and the data collection of its main trial is expected to finish in March 2013, determining any specific therapeutic effect of moxibustion might be better reserved. The benefit from the included comparative effectiveness trials may have risen from moxibustion’s specific therapeutic effect, but more likely, also from patient preferences or high expectations due to cultural backgrounds. As outcomes were all reported by participants who were not blinded, their preferences and expectations might have exaggerated the magnitude of the positive outcomes. The Rome committee for design of treatment trials of functional GI disorders also clearly pointed out that patient expectancy (placebo effect) is a major source of bias when end points are subjective [39]. Future trials may consider using pragmatic design adopting not only subjective patient-reported outcome measures but also objective outcome measures such as medication or other health service use [52].

Another issue for consideration in future practice and research is safety and economic evaluation. So far there have been few systematic reviews on adverse events of moxibustion [53]. Probable adverse events of moxibustion include allergic reactions, burns and infections [53,54]. Given the poor reporting of adverse events in the included studies, future trials should not neglect reporting adverse events in terms of incidence and severity. While acupuncture has been studied for its cost-effectiveness in various conditions [55], we have few such reports in moxibustion [56]. Due to different status of acupuncture and moxibustion treatment across countries, such data may not be applicable. It is noteworthy, however, that a recent cost-utility analysis of acupuncture as an adjunct to usual primary care for IBS reported that it could be cost-effective for patients with more severe IBS symptoms [57].

## Conclusions

This systematic review and meta-analysis suggests that moxibustion treatment has the potential for improving symptoms of IBS. We need more information; however, to firmly establish benefit-harm profile of moxibustion for IBS before we accept it as an evidence-based treatment option in our practice.

## Competing interests

The authors declare that they have no conflicts of interest.

## Authors’ contributions

Conceived of and drafted the study: HL; literature search and selection of studies: JWP, and BHL; data extraction and validation: JWP, and BHL; risk of bias assessment: JWP, and BHL; data analysis and interpretation: HL; drafting of the paper: JWP, and HL. Three authors participated actively and sufficiently in the study and approved the final manuscript.

## Pre-publication history

The pre-publication history for this paper can be accessed here:

http://www.biomedcentral.com/1472-6882/13/247/prepub

## Supplementary Material

Additional file 1 PRISMA (Preferred Reporting Items for Systematic Reviews and Meta-Analyses) checklist. Click here for file

Additional file 2 **Risk of bias assessment of the included studies.** Risk of bias was evaluated for 6 criteria in order [11], i.e. sequence generation, allocation concealment, blinding of participants, blinding of outcome assessors, incomplete outcome data, and selective outcome reporting. Each criterion was scored as yes (Y), no (N), or unclear (U), where Y indicates a low risk of bias, N indicates a high risk of bias and U indicates an unclear risk of bias. *The judgements are based on the information from the original report of individual studies and a recent Cochrane systematic review of acupuncture for IBS by Manheimer et al. [10]. AT, acupuncture; Moxa, moxibustion. Click here for file

Additional file 3 Risk of bias graph in the included trials of moxibustion for IBS. Click here for file
