# Supplementary material for: Moxibustion in the management of irritable bowel syndrome: systematic review and meta-analysis
Source: BMC Complement Altern Med. 2013 Oct 2;13:247. doi: 10.1186/1472-6882-13-247 (PMC3851749; doi:10.1186/1472-6882-13-247)
Supplement: Additional file 2 — Risk of bias assessment of the included studies. Risk of bias was evaluated for 6 criteria in order [11], i.e. sequence generation, allocation concealment, blinding of participants, blinding of outcome assessors, incomplete outcome data, and selective outcome reporting. Each criterion was scored as yes (Y), no (N), or unclear (U), where Y indicates a low risk of bias, N indicates a high risk of bias and U indicates an unclear risk of bias. *The judgements are based on the information from the original report of individual studies and a recent Cochrane systematic review of acupuncture for IBS by Manheimer et al. [10]. AT, acupuncture; Moxa, moxibustion. [file 1472-6882-13-247-S2.doc]

**Additional file 2. Risk of bias assessment of the included studies**

| **Study (year)** | **Sequence generation** | **Allocation concealment** | **Blinding of participants** | **Blinding of outcome assessors** | **Incomplete outcome data reporting** | **Selective outcome data reporting** |
| --- | --- | --- | --- | --- | --- | --- |
| **Moxibustion vs. pharmacological medications** | | | | | | |
| Luo (2012) | U  Random number generation method not described | U  Concealment method not described | N  Medication control | N  Participant assessor | Y  No missing outcome data | Y  All pre-defined outcomes reported |
| Chu (2011) | Y  Random number table used | U  Concealment method not described | N  Medication control | N  Participant assessor | Y  No missing outcome data | Y  All pre-defined outcomes reported |
| Luo (2011) | U  Random number generation method not described | U  Concealment method not described | N  Medication control | N  Participant assessor | Y  No missing outcome data | Y  All pre-defined outcomes reported |
| Luo (2008) | U  Random number generation method not described | U  Concealment method not described | N  Medication control | N  Participant assessor | Y  No missing outcome data | Y  All pre-defined outcomes reported |
| Huang (2007) | U  Random number generation method not described | U  Concealment method not described | N  Medication/colon hydrotherapy control | N  Participant assessor | Y  No missing outcome data | Y  All pre-defined outcomes reported |
| Zhang (2007) | U  Random number generation method not described | U  Concealment method not described | N  Medication control | N  Participant assessor | Y  No missing outcome data | Y  All pre-defined outcomes reported |
| Ni (2001) | U  Random number generation method not described | U  Concealment method not described | N  Medication control | N  Participant assessor | N  Insufficient reporting of number of participants analysed for improvement in global IBS symptoms outcome | N  Improvement in global IBS symptoms outcome reported incompletely so that it cannot be entered into a pooling |
| Wu (1996) | U  Random number generation method not described | U  Concealment method not described | N  Medication control | N  Participant assessor | Y  No missing outcome data | Y  All pre-defined outcomes reported |
| **Moxa/AT vs. sham moxa/AT** | | | | | | |
| Anastasi (2009) | Y  The statistician used SAS Proc Plan to  develop a scheme of randomly permuted  blocks.* | Y  Central allocation* | Y  Sham control used | Y  Blinded participant assessor | Y  Reported missing data unlikely to induce bias in effect estimate | Y  All pre-defined outcomes reported |
| **Moxa/AT vs. pharmacological medications** | | | | | | |
| Chen (2011) | Y  Random number table used | Y  Sequentially numbered, opaque and sealed envelopes used* | N  Medication control | N  Participant assessor | Y  One withdrawal from the moxa group unlikely to induce bias in effect estimate | Y  All pre-defined outcomes reported |
| Zeng (2010) | Y  Random number table used | Y  Sequentially numbered, opaque and sealed envelopes used* | N  Medication control | N  Participant assessor | Y  Reported missing data unlikely to induce bias in effect estimate | Y  All pre-defined outcomes reported |
| Xue (2009) | U  Random number generation method not described | U  Concealment method not described | N  Medication control | N  Participant assessor | Y  No missing outcome data | Y  All pre-defined outcomes reported |
| Wang (2008) | U  Random number generation method not described | U  Concealment method not described | N  Medication control | N  Participant assessor | Y  No missing outcome data | Y  All pre-defined outcomes reported |
| **Moxa plus other treatments vs. other treatments** | | | | | | |
| Hu (2012) | Y  Random number table used | U  Concealment method not described | N  Medication control | N  Participant assessor | Y  No missing outcome data | Y  All pre-defined outcomes reported |
| Shang (2012) | U  Random number generation method not described | U  Concealment method not described | N  Medication control | N  Participant assessor | Y  No missing outcome data | Y  All pre-defined outcomes reported |
| Jiang (2010) | U  Random number generation method not described | U  Concealment method not described | N  Medication control | N  Participant assessor | U  Insufficient reporting of number of participants analysed for quality of life outcome | N  Primary outcome reported using total effectiveness rate that was not pre-specified |
| Wang (2009) | U  Random number generation method not described | U  Concealment method not described | N  Medication control | N  Participant assessor | Y  No missing outcome data | Y  All pre-defined outcomes reported |
| Xiong (2008) | U  Random number generation method not described | U  Concealment method not described | N  Medication control | N  Participant assessor | Y  No missing outcome data | Y  All pre-defined outcomes reported |
| Huang (2007) | U  Random number generation method not described | U  Concealment method not described | N  Medication/colon hydrotherapy control | N  Participant assessor | Y  No missing outcome data | Y  All pre-defined outcomes reported |
| Liu (1997) | Y  Random number table used | U  Concealment method not described | N  Psychotherapy control | N  Participant assessor | N  Participant dropouts and withdrawals at follow-up were not reported | Y  All pre-defined outcomes reported |
| **Moxa/AT vs. probiotics** | | | | | | |
| An (2010) | Y  Random number table used | U  Concealment method not described | N  Medication control | N  Participant assessor | Y  No missing outcome data | Y  All pre-defined outcomes reported |
