# Supplementary figures and images for: Moxibustion in the management of irritable bowel syndrome: systematic review and meta-analysis
Source: BMC Complement Altern Med. 2013 Oct 2;13:247. doi: 10.1186/1472-6882-13-247 (PMC3851749; doi:10.1186/1472-6882-13-247)

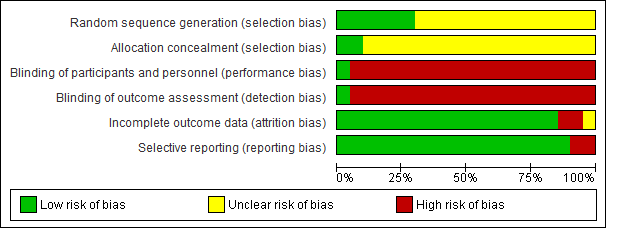

Supplement: Additional file 3 — Risk of bias graph in the included trials of moxibustion for IBS. [file 1472-6882-13-247-S3.png]
